# Psychometric evaluation of the family caregiver ICU delirium knowledge questionnaire

**DOI:** 10.1186/s12913-020-4892-5

**Published:** 2020-02-14

**Authors:** Karla D. Krewulak, Margaret J. Bull, E. Wesley Ely, Henry T. Stelfox, Kirsten M. Fiest

**Affiliations:** 1 Department of Critical Care Medicine, Alberta Health Services & University of Calgary, Calgary, AB Canada; 2 College of Nursing, Marquette University, Milwaukee, WI USA; 3 Tennessee Valley Veteran’s Affairs Geriatric Research Education Clinical Center (VA GRECC), Critical Illness, Brain Dysfunction, and Survivorship (CIBS) Center, Vanderbilt University Medical Center, Nashville, TN USA; 4 Department of Community Health Sciences and O’Brien Institute for Public Health, University of Calgary, Calgary, AB Canada; 5 Department of Psychiatry & Hotchkiss Brain Institute, Cumming School of Medicine, University of Calgary, Calgary, AB Canada

**Keywords:** Delirium prevention and management, Family caregivers, Critical care, Intensive care unit

## Abstract

**Background:**

Delirium is a common condition in critically ill patients, affecting nearly half of all patients admitted to an intensive care unit (ICU). Family caregivers of critically ill patients can be partners in the early recognition, prevention and management of delirium provided they are aware of the signs/symptoms and appropriate non-pharmacological strategies that might be taken. Valid, reliable instruments that assess family caregiver knowledge are essential so that nurses can prepare family caregivers to be effective partners. The purpose of the current study was to (a) adapt an existing caregiver delirium knowledge questionnaire (CDKQ) for use by nurses to measure a family caregiver’s delirium knowledge in the ICU; and (b) examine the psychometric properties and structure of the adapted Caregiver ICU Delirium Knowledge Questionnaire (CIDKQ).

**Methods:**

In this cross-sectional study, a multidisciplinary team developed the 21-item CIDKQ (possible score range: 0–21) and administered it to 158 family caregivers of critically ill patients. Descriptive statistics were examined for all variables. The CIDKQ was analyzed for face validity, content validity, reliability and internal consistency.

**Results:**

The mean CIDKQ score was 14.1 (SD: 3.5, range = 2 to 21). Path analysis revealed that a family caregiver’s delirium knowledge in the actions and symptoms dimensions had a direct effect on knowledge of delirium risk factors. The CIDKQ was found to have face validity and reliability (Cronbach’s α = 0.79).

**Conclusions:**

The findings indicated good validity and reliability of the CIDKQ as a measure of ICU delirium knowledge in family caregivers of critically ill patients.

## Background

Delirium is a common neuropsychiatric complication in the ICU that affects over 50% of critically ill patients [1–3] and is associated with negative short-term (e.g., longer ICU stay, increased ICU and in-hospital mortality) and long-term outcomes (e.g., post-intensive care syndrome, (PICS)) [4]. Family caregivers often experience distress from witnessing delirium in their loved ones [5–8], which can lead to the development of adverse psychological outcomes after ICU discharge known as PICS-Family [9–11]. The Society of Critical Care Medicine guidelines for family-centred care in the ICU [12] suggest providing family education programs and including family caregivers in patient care has the potential to improve family caregiver-centred outcomes [10, 12]. Current delirium guidelines recommend multicomponent non-pharmacological interventions such as a reorientation protocol (eyeglass/hearing optimization, orientation of day/time/location), environment protocol (improve sleep) or mobility have the potential to prevent or reduce the number of delirium days [13]. These non-pharmacological interventions can be employed by family caregivers, providing an opportunity for family caregivers to be engaged in patient care.

Many family caregivers do not possess sufficient delirium knowledge to function as partners in the prevention and management of delirium using non-pharmacological strategies. A valid, reliable caregiver delirium knowledge questionnaire (CDKQ) to help nurses evaluate a family caregivers’ delirium knowledge exists, but was validated in community-dwelling family caregivers of elective knee and hip patients [14]. There are additional risk factors that predispose a patient to develop delirium in the ICU (e.g., severity of illness, mechanical ventilation, use of benzodiazepines) [13, 15] compared to that of a patient in the hospital or community. To our knowledge, a valid, reliable measure of family caregiver knowledge of ICU delirium does not exist in the literature. The purpose of this study was to (a) adapt the existing CDKQ for use in family caregivers of critically ill patients in the ICU and (b) establish the validity and reliability of this Caregiver ICU Delirium Knowledge Questionnaire (CIDKQ). In turn, nurses can use the CIDKQ to specifically target learning needs of family caregivers and better prepare them to partner in the prevention and management of delirium. This might improve patient- and family-centred outcomes.

## Methods

### Instrument refinement

The CDKQ questions were reworded to be ICU-specific. After reviewing relevant literature on ICU delirium risk factors and outcomes, one additional item (increased delirium risk in patients who are mechanically ventilated) was added to the CIDKQ [13, 15, 16]. Similar to the CDKQ [14], the Symptom Interpretation Model (SIM) framework was used [17], which includes three dimensions of delirium knowledge: risks, action and symptoms. A total of 21 multiple choice (yes/no/don’t know) items were included in the CIDKQ addressing three dimensions of delirium knowledge: risk factors (items 1–10), actions (items 11–16) and symptoms (items 17–21). Any correct answer is coded as 1 and any incorrect answer (including “don’t know”) as 0.

#### Design and sample

The study was approved by the Conjoint Health Research Ethics Board at the University of Calgary (Reference number: REB18–0331). To validate the CIDKQ, we performed a cross-sectional study between June 19, 2018 and July 31, 2019 using a convenience sample of family caregivers from the 28-bed general systems adult ICU and the family waiting rooms for the 16-bed cardiovascular ICU (CV-ICU) at Foothills Medical Centre in Calgary, Canada (catchment population: 1.5 million). The general systems adult ICU includes neurological, medical, surgical and long-term ICU patients. Upon ICU admission, patients and their families are provided with an ICU delirium pamphlet as part of a welcome package. The CV-ICU includes patients who are pre- and post-cardiovascular surgery. Bedside nurses screen for delirium once per shift (twice daily) in both the ICU and CV-ICU. The prevalence of delirium in 14 Alberta ICUs between January 2014 and June 2016 was 56.7% (95% Confidence Interval: 55.8–57.5) (Fiest KM, Soo, A, Lee, CH., Niven, DJ, Ely, EW, Doig, CJ, Stelfox, HT: Long-Term Outcomes of Delirium in Patients Admitted to the Intensive Care Unit: A Multi-Centre, Population-Based Cohort Study, in preparation).

We followed the Strengthening the Reporting of Observational Studies in Epidemiology (STROBE) guidelines for cross-sectional studies [18]. Inclusion criteria include: adults ≥18 years of age who could provide written, informed consent and understand the English language. For the purpose of this study, a family caregiver was considered to be anyone who met this inclusion criteria. A research assistant approached eligible participants and used a script that included a standardized description of delirium. Participating family caregivers completed a paper version of a demographic questionnaire (relationship to patient, date of birth, sex, gender, ethnicity and educational background) and a paper version of the CIDKQ, both completed after enrollment in the presence of the study team.

#### Sample size

Sample size for the path analysis was calculated using the general rule of 10–20 participants per estimated parameter [19]. In our path analysis, we had two regressions and four covariances, totaling six parameters. For a ratio of 10–20 participants per estimated parameter (six), we required a sample size between 60 and 120.

#### Data analysis

Descriptive statistics were examined for all study variables. Results were expressed in frequencies and percentages for categorical variables or through mean and standard deviation for continuous variables.

### Validity and reliability of the CIDKQ

As there is no gold or reference standard for measuring family caregiver ICU delirium knowledge, the validity of the CIDKQ was determined through face validity and construct validity. A critical review of the CIDKQ items was discussed with a multidisciplinary group including delirium researchers, patient partners (past ICU patients and family members), ICU physicians, ICU nurses and allied health professionals. ICU nurses and allied health professionals were part of a delirium working group that meets monthly to discuss delirium initiatives. ICU physicians and patient partners are part of the department and research team, respectively. Any, additional items were discussed with the same multidisciplinary team to ensure items were simple, clear and related to ICU delirium knowledge. Due to the sample size, determination of construct validity was not possible using confirmatory factor analysis as was used in the original study of the CDKQ development [14]. Instead, we tested the model from the correlation of the three dimensions of delirium knowledge: risks (items 1–10, score range 0–10), actions (items 11–16, score range 0–6) and symptoms (items 17–21, score range 0–5). We used Pearson correlation coefficients to examine the correlation between the dimensions of delirium knowledge, wherein a Pearson r value of 0.10, 0.30, and 0.50 was interpreted as small, medium, and large effect sizes, respectively [20]. We used path analysis to examine direct effects of the dimensions of delirium knowledge using maximum likelihood estimation. Path coefficients including the observed endogenous variables were calculated using the Stata package pathreg [21] with Stata version 14 (StataCorp., College Station, TX, USA).

The reliability of the CIDKQ was determined by measuring internal consistency (Cronbach’s alpha). Cronbach’s alpha was calculated using Stata (StataCorp, College Station, TX) wherein a correlation of 0.7 was considered good and 0.8 considered excellent [22].

## Results

**Fig. 1:**
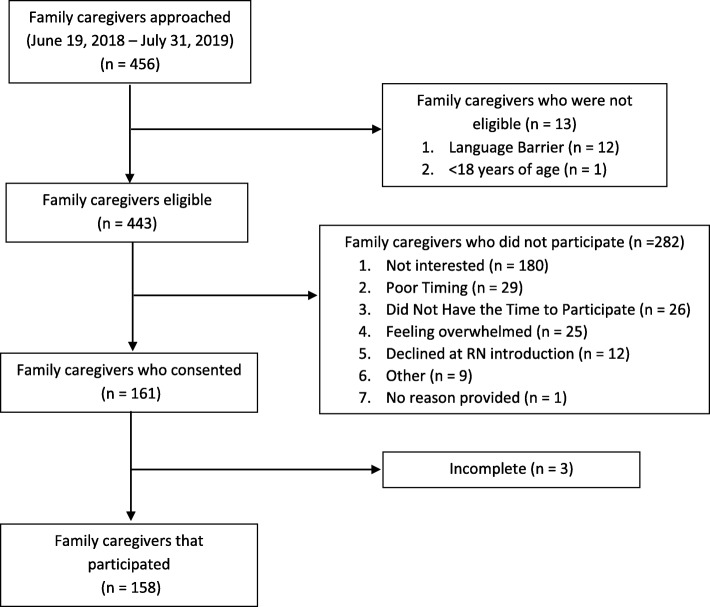
Flow diagram showing family caregiver participation

Between June 2018 and July 2019, a total of 158 participants from the CV-ICU (33/158, 20.9%) and ICU (125/158, 79.1%) completed the CIDKQ, with a participation rate of 35.7% (158/443) (Fig. 1). Completion of the questionnaire took three to five minutes and no participant indicated it was a burden to complete. Participant characteristics are listed in Table 1. Mean participant age was 47.6 years (standard deviation [SD] 15.4 years). The majority of participants were female (97/158, 61.4%), adult children (40/132, 30.3%) of the patient. A total of 3300 answers (out of a possible 3318) were recorded for the CIDKQ, with 18 answers missing at random (18/3318, 0.5%). The mean score of the CIDKQ was 14.2 (SD: 3.4, range = 2 to 21) (Table 1). The subscale means were 6.5 (SD: 1.9, range 0–10, possible range 0–10) for the risk, 4.2 (SD: 1.3, range 0–6, possible range 0–6) for the action and 3.5 (SD: 1.2, range 1–5, possible range 0–5) for symptom recognition subgroups. The items answered correctly by the least number of family caregivers were “If your family member had signs of sudden confusion, would you let the patient sleep during the day?” (action subscale) and items 17 and 18 of the symptom recognition subscale (“Patient slowly becomes more confused over a few months (prior to ICU admission) …” )(Table 2).

**Table 1:** Demographics of the Study Sample (*n* = 158)

Characteristics	n (%)
Type of Family Caregiver:	
Child	44 (27.8)
Other	34 (21.5)
Spouse	33 (21.0)
Sibling	29 (18.3)
Parent	18 (11.4)
Education:	
High school or less	52 (32.9)
More than a high school	101 (63.9)
Not provided	5 (3.2)
^a^Cultural group/race:	
Not provided	70 (44.3)
Caucasian/White	32 (20.3)
European	22 (13.9)
North American	15 (9.5)
Asian	10 (6.3)
First Nations	3 (1.9)
Metis	3 (1.9)
Indian/African	2 (1.2)
Black	1(0.6)
Female gender	97 (61.4)
Female sex	97 (61.4)
Age, mean ± standard deviation (range)	47.6 ± 15.4 (18–81)
CIDKQ score, mean ± standard deviation (range)	14.2 ± 3.4 (5–21)

^a^Participants were asked to self-identify their ethnicity, culture, race in a free-text box. They were not required to provide an answer

**Table 2:** Correctly answered items for the Caregiver ICU Delirium Knowledge Questionnaire (CIDKQ) (n = 158)

Item number	Description	Correct answer	Correctly answered, n (%)
Risk subgroup			
Do you think any of the patients below might be at risk for delirium?			
1	Patients who are older	Yes	121 (77.1)
2	Patients who are married (vs. not married)	No	74 (47.4)
3	Patients with dementia	Yes	129 (81.7)
4	Patients with an infection	Yes	132 (84.1)
5	Patients with more than high school education	No	80 (51.3)
6	Patients who had recent surgery	Yes	137 (86.7)
7	Patients who are dehydrated	Yes	121 (77.1)
8	Patients experiencing change in surroundings such as admission to a hospital or change to another part of the hospital	Yes	115 (73.2)
9	Patients who are mechanically ventilated or intubated	Yes	119 (75.3)
10	Patients started on a new medication	Yes	112 (71.3)
Action subgroup			
If your family member had sign of sudden confusion, would you:			
11	Orient patient to time and day and bring in photos from home	Yes	126 (80.2)
12	Wait 24 h to see if the person got better	No	82 (51.9)
13	Let the patient sleep during the day to recover	No	49 (31.8)
14	Do nothing	No	146 (92.4)
15	Inform the bedside RN or another member of the care team right away	Yes	149 (94.9)
16	Ask the care team about medication changes	Yes	137 (86.7)
Symptom subgroup			
Do you think any of the patients described below might have delirium?			
17	Patients slowly becomes more confused over a few months is forgetful has trouble paying attention and is more confused later in the day	No	38 (24.0)
18	Patients slowly becomes more confused over a few months is forgetful has trouble paying attention and later in the day sees things that are not there	No	19 (12.1)
19	Patients suddenly becomes confused over a few days or hours floats in and out of confusion during the day has trouble paying attention sees things that are no there	Yes	138 (87.3)
20	Patients suddenly becomes confused over a few days or hours has trouble paying attention and sleeps more during the day	Yes	116 (73.9)
21	Patients becomes more confused over a few days and suddenly has trouble getting to the bathroom on time	Yes	93 (59.2)

### Validity

Based on the ICU delirium literature and ICU delirium experts’ recommendations, several changes were made to the CDKQ to adapt it for the ICU setting (Additional file 1: Table S1). The wording “older adult” in the CDKQ was changed to “patients” for the CIDKQ. For the risk subgroup, the age for increased delirium risk was changed from “adults older than 70” to “patients who are older.” The question about race or biological sex as a risk for delirium were removed because there is no consensus in the ICU delirium literature associating either with increased ICU delirium risk [15]. Lastly, an additional risk factor was added: increased delirium risk in patients who are mechanically ventilated [15, 23]. The action subgroup included additional questions suggested by the authors of the CDKQ (“Orient patient to time of day and bring in photos from home” and “Ask the care team about medication changes”), which are supported by a narrative review on non-pharmacologic treatment and medication minimization [24]. One additional item, “Let the patient sleep during the day to recover” was added to encourage family caregivers to assist with a day/night routine as a non-pharmacological strategy to prevent or manage delirium. No items were changed in the symptoms subgroup as they represent a range of ICU patients and address dementia versus delirium. There was 100% agreement from the experts on the content and placement of the additional questionnaire items. Members of the multidisciplinary team found all questionnaire items were simple, clear and related to ICU delirium knowledge.

**Fig. 2:**
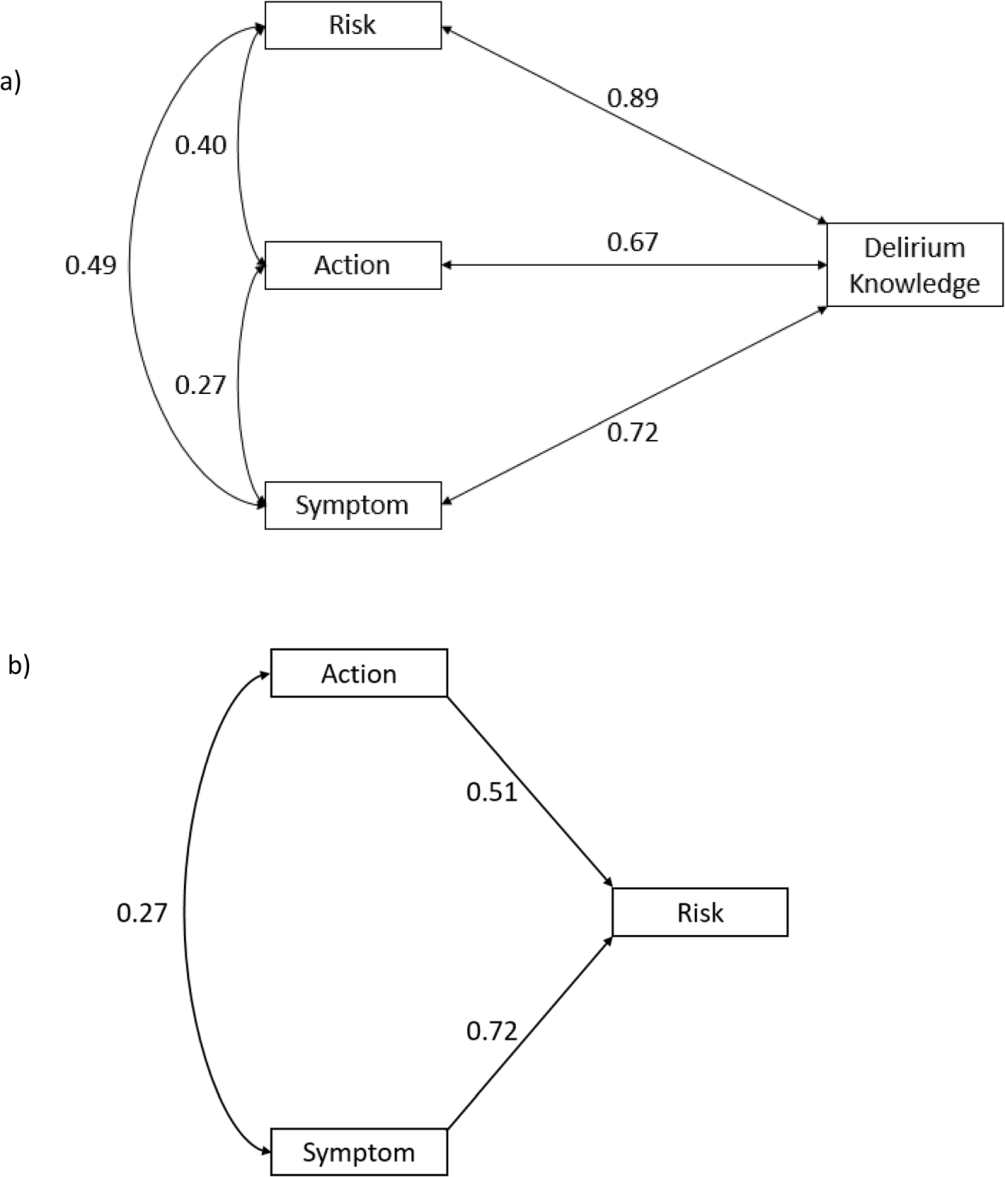
**a** Pearson correlation coefficients between each of the three dimensions of delirium knowledge (risk, actions and symptoms) and delirium knowledge (represented by total Caregiver ICU Delirium Knowledge Questionnaire score). **b** Proposed pathway between the dimensions of delirium knowledge (risk, actions and symptoms). Single- and double-headed arrows represent directional effects from one variable to another and correlations between variables, respectively. Numbers on each arrow represent the Pearson correlation coefficient (double-headed arrow) and standardized path coefficient (single-headed arrow)

Figure 2 shows the Pearson correlation coefficients between each dimension of delirium knowledge and the proposed pathway that had the best fit between the three dimensions of delirium knowledge (risks, actions and symptoms). The correlations ranged from small (0.27, correlation between the risk and action dimensions) to medium (0.40 and 0.49 between risk and action dimension and action and symptom dimension, respectively). All correlations between the risk, action or symptom dimension and total CIDKQ score were considered large. An increase in total action and total symptom scores are significantly associated with an increase in total risk score (Fig. 2).

### Reliability

Cronbach’s alpha for internal consistency of the CIDKQ was 0.79 for the total scale, which indicates the CIDKQ has good reliability. Cronbach’s alpha were 0.77 for the risk subscale, 0.43 for the action subscale and 0.57 for the symptom recognition subscale. Examination of the Cronbach’s alpha for each item indicates that the total Cronbach’s alpha did not change if any items were deleted from the CIDKQ (Table 3). However, items 16 and 18 had have a low correlation with the total score. If both items are removed, the Cronbach’s alpha is 0.80.

**Table 3:** Item analysis for the family caregiver ICU delirium knowledge questionnaire

Item number	Description	Alpha if item deleted	Item-to-total score correlations
1	Patients with an infection	0.78	0.54
2	Patients who are married (vs not married)	0.80	0.33
3	Patients with dementia	0.78	0.54
4	Patients who are older	0.78	0.56
5	Patients with more than a high school education	0.79	0.37
6	Patients who had recent surgery	0.78	0.61
7	Patients who are dehydrated	0.77	0.70
8	Patients experiencing change in surroundings such as a recent ICU admission or move to new room or transferred	0.77	0.69
9	Patients started on a new medication	0.78	0.51
10	Patients who are mechanically ventilated or intubated	0.79	0.48
11	Orient patient to time and day or bring in photos from home	0.79	0.38
12	Wait 24 h to see if the patient got better	0.79	0.40
13	Let the patient sleep during the day to recover	0.80	0.33
14	Do nothing	0.79	0.32
15	Inform the bedside RN or another member of the care team right away	0.79	0.39
^a^16	Ask the care team about medication changes	0.80	0.23
17	Patient slowly becomes more confused over a few months (prior to ICU admission), is forgetful, has trouble paying attention, and is more confused later in the day	0.80	0.30
^a^18	Patient slowly becomes more confused over a few months (prior to ICU admission), is forgetful, has trouble paying attention and later in the day sees things that are not there	0.80	0.23
19	While in the ICU, the patient suddenly becomes confused over a few days or hours, floats in and out of confusion during the day, has trouble paying attention, sees things that are not there	0.78	0.56
20	While in the ICU, the patient suddenly becomes confused over a few days or hours, has trouble paying attention, and sleeps more during the day	0.78	0.49
21	While in the ICU, the patient becomes more confused over a few days and suddenly has trouble getting to the bathroom on time	0.79	0.43

^a^Items with low correlation and can be removed from the CIDKQ

## Discussion

ICU nurses evaluating a family caregiver’s delirium knowledge and educating family caregiver’s based on their knowledge gaps is a vital step for family caregivers to partner effectively in the prevention and management of delirium. This study demonstrated that the CIDKQ is a valid and reliable tool to measure a family caregiver’s knowledge of the three dimensions of ICU delirium knowledge: risks, actions, symptoms. The content of the CIDKQ was based on relevant ICU delirium literature and consensus among a multidisciplinary team.

The reliability of the CIDKQ is comparable to the CDKQ [14]. The number of items in the risk subscale was the same (*n* = 10), but the internal consistency was better in the CIDKQ (0.77) for the risk subscale versus the CDKQ (0.66). Even though the CIDKQ actions subscale had two more items (*n* = 6) than the CDKQ (*n* = 4), the Cronbach alpha for the actions subscale was lower than the CDKQ (0.43 versus 0.72). The symptom subscale had the same number of items and a higher Cronbach alpha (0.57) compared to the CDKQ symptom subscale (0.49). Items in the CIDKQ include with low correlation are items 16 and 18, which include “Ask the care team about medication changes” and description of a patient with dementia. As there is already a dementia question (item 17), item 18 can be removed. Although item 16 has low correlation with the overall score, it should remain because it empowers a family caregiver to ask the care team about possible deliriogenic medications such as benzodiazepines or prompt a discussion with the care team about their loved one’s altered cognition which may be secondary to analgesia or sedation administration (i.e. not delirium). Further refinement of the CIDKQ could include additional risk factors such as patients with hypertension, patients in a coma [15] or sleep disturbance [25]. Additional items added to the actions and symptoms subscales, which may improve the reliability and the fit indices, include non-pharmacological interventions from the current delirium guidelines [13] such as “bring in eyeglasses or hearing aids” for the action subscale and an item describing a patient with agitation for the symptom subscale.

The CIDKQ has applicability in clinical practice. First, it allows for ICU nurses to assess a family caregiver’s knowledge of delirium risk factors, appropriate actions when a patient has delirium and delirium symptoms and if they can distinguish delirium from dementia. As such, the ICU nurse can target delirium education to the knowledge gaps and empower the family caregiver to partner effectively to prevent and manage delirium. Family caregivers are an underutilized resource and are well-positioned to partner in prevention and management delirium. Family caregivers know the patient the best and can relate to the patient in a way that an unfamiliar member of the healthcare team cannot. This includes applying nonpharmacological strategies tailored to the patient’s known preferences or calming a patient who is agitated. A recent systematic review reports that family caregiver involvement in delirium may reduce a family caregiver’s anxiety and reduces a patient with delirium length of hospital stay [26]. In addition, most physicians and nurses believe families can assist with delirium prevention [27] and do not view providing delirium education to family caregivers as onerous [28]. Family caregivers are accepting of providing interventions to patients with delirium [26].

### Limitations

The current study followed established cross-sectional study reporting guidelines and included a multidisciplinary team. However, there are still a few limitations. The study was conducted in two ICUs located within a single centre hospital. FMC has a catchment area of 1.5 million and includes a diverse population. On patient admission, the sampled ICU provides a pamphlet on delirium (with information on delirium risk factors, actions and symptoms), regularly screens for delirium using the Intensive Care Delirium Screening Checklist [29] and reports delirium as part of daily, multidisciplinary rounds, which family caregivers are invited to participate in. As such, family caregivers in this sample may have better delirium knowledge and these results may not be generalizable to family caregivers from other ICUs.

## Conclusions

The CIDKQ is a valid and reliable measure of delirium knowledge of family caregivers of critically ill patients. This measure can help ICU nurses and researchers identify the need for delirium education in family caregivers of critically ill patients and evaluate the effectiveness of that education. The CIDKQ can be adapted for other ICU settings and updated and re-validated to align with future guideline updates.

## Supplementary information

**Additional file 1: Table S1**. Comparison of the items of the Caregiver Delirium Knowledge Questionnaire (CDKQ) and Caregiver ICU Delirium Knowledge Questionnaire (CIDKQ)

## Data Availability

The questionnaire and data used during the current study are available from the corresponding author (KMF) on reasonable request.

## Abbreviations

CDKQ: Caregiver Delirium Knowledge Questionnaire
CIDKQ: Caregiver ICU Delirium Knowledge Questionnaire
CV-ICU: Cardiovascular Intensive Care Unit
ICU: Intensive Care Unit
PICS: Post-Intensive Care Syndrome
