# Supplementary material for: Psychometric evaluation of the family caregiver ICU delirium knowledge questionnaire
Source: BMC Health Serv Res. 2020 Feb 14;20:116. doi: 10.1186/s12913-020-4892-5 (PMC7023729; doi:10.1186/s12913-020-4892-5)
Supplement: Supplementary file 1 — Additional file 1: Table S1. Comparison of the items of the Caregiver Delirium Knowledge Questionnaire (CDKQ) and Caregiver ICU Delirium Knowledge Questionnaire (CIDKQ) [file 12913_2020_4892_MOESM1_ESM.docx]

**Table S1.** Comparison of the items of the Caregiver Delirium Knowledge Questionnaire (CDKQ) and Caregiver ICU Delirium Knowledge Questionnaire (CIDKQ)

| **Item** | **CDKQ** | **Item** | **CDKQ** |
| --- | --- | --- | --- |
| 1 | Adults older than 70 | 1 | Patients who are older |
| 2 | Older adults who are married | 2 | Patients who are married (vs. not married) |
| 3 | Older adults with dementia | 3 | Patients with dementia |
| 4 | Older adults with an infection | 4 | Patients with an infection |
| 5 | Older adults with more than high school education | 5 | Patients with more than high school education |
| 6 | Older adult who has had surgery | 6 | Patients who had recent surgery |
| 7 | Older adults not drinking enough liquid (becoming dehydrated) | 7 | Patients who are dehydrated |
| 8 | Older adults experiencing change in surroundings, such as a move to home or hospital | 8 | Patients experiencing change in surroundings such as admission to a hospital or change to another part of the hospital |
| 9 | Older adults who are members of minority groups | 9 | Patients who are mechanically ventilated or intubated |
| 10 | Older adults started on a new medication | 10 | Patients started on a new medication |
| 11 | Call their doctor right away | 11 | Orient patient to time and day and bring in photos from home |
| 12 | Wait a week to see if the person got better | 12 | Wait 24 hours to see if the person got better |
| 13 | Give the person herbal tea of warm milk to drink | 13 | Let the patient sleep during the day to recover |
| 14 | Do nothing | 14 | Do nothing |
| 15 | Patients slowly becomes more confused over a few months is forgetful has trouble paying attention and is more confused later in the day | 15 | Inform the bedside RN or another member of the care team right away |
| 16 | Patients slowly becomes more confused over a few months is forgetful has trouble paying attention and later in the day sees things that are not there | 16 | Ask the care team about medication changes |
| 17 | Patients suddenly becomes confused over a few days or hours floats in and out of confusion during the day has trouble paying attention sees things that are no there | 17 | Patients slowly becomes more confused over a few months is forgetful has trouble paying attention and is more confused later in the day |
| 18 | Patients suddenly becomes confused over a few days or hours has trouble paying attention and sleeps more during the day | 18 | Patients slowly becomes more confused over a few months is forgetful has trouble paying attention and later in the day sees things that are not there |
| 19 | Patients becomes more confused over a few days and suddenly has trouble getting to the bathroom on time | 19 | Patients suddenly becomes confused over a few days or hours floats in and out of confusion during the day has trouble paying attention sees things that are no there |
|  |  | 20 | Patients suddenly becomes confused over a few days or hours has trouble paying attention and sleeps more during the day |
|  |  | 21 | Patients becomes more confused over a few days and suddenly has trouble getting to the bathroom on time |
